# Protocols to Study Aging in *Drosophila*

**DOI:** 10.1007/978-1-4939-6371-3_18

**Published:** 2016-10-12

**Authors:** Matthew D. W. Piper, Linda Partridge

**Affiliations:** 3 Institute of Healthy Ageing and Department Genetics, Evolution and Environment, University College London, Gower St, London, UK; 4 Max Planck Institute for Biology of Ageing, Joseph-Stelzmann-Str. 9b, D-50931 Köln, Germany; 5 School of Biological Sciences, Monash University, Clayton, Victoria 3800 Australia

**Keywords:** *Drosophila melanogaster*, Life span, Aging, Method, Diet, Genetic interventions, Pharmacological interventions, Backcross

## Abstract

The fruit fly *Drosophila melanogaster* offers a host of advantages for studying the biology of aging: a well-understood biology, a wide range of genetic reagents, well-defined dietary requirements, and a relatively short life span, with a median of ~80 days and maximum ~100 days. Several phenotypes can be used to assess the aging process, but the simplest and most widely used metric is length of life. Here we describe a standard life span assay for *Drosophila* housed on a simple sugar/yeast diet.

## Introduction

As Aging Drosophilaaround the world age, increasing effort is being devoted to the Development of new approaches to improve the health of older people. Remarkably, experimental work on worms, flies and mice over the last 20 years has provided a positive outlook on this prospect [1, 2] For these model organisms, genetic, environmental, and Pharmacological interventions have been described that extend healthy Life span [3]. Even more remarkably given the very different life spans of these model organisms, these interventions often act on common mechanisms to extend life span, implying some degree of evolutionary conservation of mechanisms of aging. Thus there is great promise that studies of aging in laboratory model organisms will yield insights into aging that will ultimately benefit humans.

The challenges of experimental gerontology are enormous. Experiments require long time-scales, genetic manipulations, large populations, and well-controlled animal stocks and conditions. These factors make the work perfectly suited to the small, short-lived, and well-characterized model organisms such as the fruit fly Drosophila melanogaster.

Life span experiments have been conducted on *Drosophila* for the last 100 years [4] and over time the conditions have been refined [5]. In general, the protocol can be simple, but small and seemingly insignificant modifications to experimental protocols can have large effects on outcomes. For example, by not controlling for Diet quality, genetic background or the interactions between mating frequency and Diet the experiment may report the effects on Life span of an uncontrolled, trivial experimental procedure, rather than the focal intervention of the study [6].

Here we outline the basic procedure for rearing, isolating, and maintaining flies for Life span experiments, highlighting a number of the known pitfalls that have misled researchers in the past. We provide a basic protocol for wild type flies housed under our standard laboratory conditions and then we provide modified protocols for studying the effects on life span of Diet, drugs, or Genetic interventions.

## Materials

### Media

All media are prepared using reverse osmosis water. Cooking can be done on a gas hob using a standard saucepan and stirring with a heavy-duty whisk (*see* **Note** **1**).

1. Egg collection medium (volume sufficient for ~10 × 15 cm petri dishes): to 250 ml cold water add 12.5 g agar and stir to mix. Bring to boil while stirring and maintain boiling for ~2 min to ensure agar is completely melted. Add 150 ml red grape juice (*see* **Note** **2**) and stir until the mixture returns to the boil. Remove from heat. Add 25 ml cold water and stir until temperature drops to ~65 °C. Make 10.5 ml 10 % Nipagin (methyl 4-hydroxybenzoate in 95 % ethanol) and pour solution into petri dishes. Allow to cool at room temperature, allowing steam to escape. Ensure to protect the plates from any flies at this stage to avoid contamination. Cover and store at 4 °C.
2. Fly food for rearing and maintenance (makes 1 L of 1SY [7], *see* **Note** **3**): add 15 g agar to 700 ml cold water and stir. Heat until boiling. While continuing to stir, add 50 g table sugar (sucrose) and 100 g yeast (whole yeast autolysate and not water soluble yeast extract). After returned to boil, remove from heat and add cold water to make up to final volume of 1 L. Stir and allow to cool to ~65 °C. Mix in 30 ml 10 % nipagin and 3 ml propionic acid to act as preservatives. This is also the point at which to mix in any small volume additions of drugs/transgene inducers/vehicle control. For larger volume additions, reduce the cold water addition after cooking to ensure final total volume remains at 1 L. Using a peristaltic pump with clean, sterilized tubing, dispense into clean vials or bottles. Allow to cool at room temperature for several hours (*see* **Note** **4**). To avoid contamination, ensure to protect cooling food from flies (*see* **Note** **5**). Plug individual vials with cotton balls (*see* **Notes** **6** and **7**). Store at 4 °C.
3. Live yeast paste for stimulating egg laying: Mix dried baker’s yeast granules with cold water at a ratio of approx. 1:1 by Weight to make a stiff paste (ice cream consistency). Best when used immediately, but can be stored covered at 4 °C for 2 weeks.

### Plastic/Glassware for Housing and Handling Eggs and Flies (**See***Note***8**)

1. 15 cm diameter plastic petri dishes (*see* **Note** **9**).
2. Fly “cage” for housing parental flies: an ~15 cm long plastic cylinder that fits a petri dish snugly at one end, and is covered with mesh at the other.
3. Bottles: ~250 ml (polypropylene or glass; ~60 mm OD × 130 mm H).
4. Vials/tubes: ~15 ml (polystyrene, polypropylene or glass; ~ 25 mm O.D. × 95 mm H).
5. Cotton wool balls or high density synthetic bungs to close tubes.
6. Squeeze bottle.
7. Pipette (20–100 μl).
8. Wide bore pipette tips (**Note** **10**).
9. CO_2_ stream—supplied via a water bubbler and low-static porous diffusion pad.
10. Fine paint brush (size 000–0000).
11. Handle-mounted metal pick.

### Solutions for Handling Eggs and Flies

1. Phosphate buffered saline. Mix pre-formulated tablets with water according to instructions on container. This yields 0.01 M phosphate buffer, 0.0027 M potassium chloride, 0.137 M sodium chloride, pH 7.4.

## Methods

### Parental Generation

#### Preparing Stocks for Egg Collection

Most laboratory stocks are kept in small numbers and under crowded conditions, both of which alter adult Life span [6, 8].

It is important to implement procedures to control these factors so that they do not confound interpretations of alterations in fly Life span

1. “wild types”: to escape the transgenerational effects of stock crowding on life span, we passage stock-derived flies through two generations of our standard density procedure before use in life span experiments.
2. Genetic crosses: it is extremely important to standardize the genetic background of all mutant lines to be compared in a Life spanexperiment. Failure to do so is common and leads to incorrect conclusions about the effects of Genetic interventions to extend life. Most experimental transgenic flies are generated by crossing two inbred lines, with one containing the transgene to be activated and the other containing a genetic construct that drives the expression of the first. This cross also produces a hybrid genetic background, and this will generally increase Life span when compared with that of the inbred controls, as a consequence of heterosis and irrespective of any effect of the transgenes [9]. To avoid this problem, all transgenes and mutants should first be backcrossed into a standardized genetic background for at least six generations. To maintain the lines an additional 2–3 Backcrosses should be repeated every 6–12 months (*see* **Note** **11**). Furthermore, each of the transgenic lines used to construct the experimental line should be included as a control in the Life span experiment, because transgenes can cause insertional mutagenesis, which can in turn modify longevity.

#### To Collect Staged Embryos

1. House parental flies in “cages.” Provide a generous smear of live yeast paste (~1 tsp) at the center of the egg laying plate.
2. After 48 h, replace egg laying surface (*see* **Note** **12**) with a fresh plate harboring a fresh aliquot of live yeast paste (egg laying peaks ~72 h after introduction to rich food) (*see* **Note** **13**).
3. Leave overnight (*see* **Note** **14**).
4. Collect embryos for Development at standard density. To achieve this, we either use a pipette to allocate a fixed volume of a dense embryo suspension into new media for Development, or use a mounted metal pick to collect and transfer individual larvae to development media (*see* **Note** **15**):

*Pipetting* Method *(ideal for robust* Genotypes *, to yield large numbers of experimental flies)*:

1. Anesthetize flies in cage, remove egg laying plate on which fertilized eggs lie and discard any yeast paste not consumed (*see* **Note** **16**).
2. Using a squeeze bottle containing PBS, cover the plate with a thin layer of buffer.
3. Dislodge eggs by “brushing” the egg laying surface with a fine paint brush.
4. Pour egg/PBS suspension into a 15 ml falcon tube and allow eggs to settle.
5. Pour off most of the PBS and add more fresh PBS to wash the eggs.
6. Allow eggs to settle and pour off most of the PBS, leaving only sufficient to cover the settled egg mass.
7. Allow eggs to settle.
8. Using 100 μl pipette with wide bore tip (*see* **Note** **10**), set volume to 18–20 μl and insert tip into the solution so the tip is level with the top of settled egg mass; quickly release plunger while dropping tip into the mass of eggs.
9. Inspect tip for a dense, even, mass of eggs (*see* **Note** **17**).
10. Dispense egg mass on to surface of ~70 ml SY medium in a 250 ml bottle.

*Picking* Method *(more labor-intensive than pipetting, but more fragile* Genotypes *tend to fare better using this* Method):

1. Incubate egg laying plate with staged embryos for 24 h at 25 °C (*see* **Note** **18**)
2. Using a dissecting microscope, locate first instar larvae (*see* **Note** **19**) on plate and touch with the metal pick. They will stick.
3. With practice, up to ~20 larvae can be collected on one pick.
4. Gently transfer picked larvae into a fresh vial with food for Development by wiping larvae off the needle on to the surface of the food (*see* **Notes** **20** and **21**)
5. Plug vial/bottle tightly with cotton wool ball(s) (*see* **Note** **22**)
6. Incubate at 25 °C with 65 % humidity and 12:12 h light–dark

### Experimental Generation

#### Measuring Life Span of One Batch of Mated Wild Type Flies on One Food Type

**Fig. 1:**
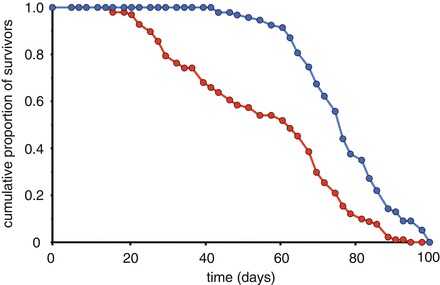
Examples of good and poor quality survival data. The survival characteristics of a healthy population of flies are demonstrated in *blue*. There are relatively few deaths up until day 60, from which point there is rapid loss of life. By contrast, the population illustrated by the *red line* suffers substantial numbers of deaths beginning at day 20. Thus many flies are dying at young and middle ages, rather than predominantly at old age. This is a sign of poor housing conditions or a genetically fragile stock

1. After 10 days, transfer freshly emerged flies to fresh bottles or vials containing SY medium (*see* **Notes** **23** and **24**)
2. Return bottles of flies to controlled environment (25 °C, 65 % humidity and 12:12 h light–dark) for 48 h to allow all flies to mate
3. Anesthetize flies with CO_2_ and manipulate carefully using a soft brush (*see* **Note** **25**)
4. Separate males from females and allocate target individuals to experimental containers (refer to **Notes** **26**–**30**). Various aspects of Courtship and mating modify Life span of the different sexes to different extents [10–14]. Also, Genotype and food quality interact with Courtship and mating frequency [15, 16]. Housing experimental flies as a single sex population avoids the confounding effects of sex X treatment interactions that modify Life span.
5. Store vials at 25 °C, 65 % humidity, 12:12 h light–dark (refer to **Note** **31**)
6. Transfer flies to fresh food every 2–3 days (refer to **Notes** **32** **–** **35**)
  - If recording female egg laying of experimental flies, a good and simple summary can be generated by counting all eggs in all vials once or twice a week for the first 4–5 weeks
    Count the number of eggs on the surface of the food ~18 h after transferring in flies (*see* **Notes** **36**–**38**)
    Data for a vial are expressed as the number of eggs per fly per day. For each vial, sum the egg lay for the average female across all count days to generate an index of lifetime egg laying.
7. For each transfer, score deaths and censors until all flies are dead (*see* Fig. 1 for survival data examples).

#### Protocol Modifications for Measuring the Effects of Dietary Interventions

1. Repeat **steps 1**–**3** from Subheading 3.2.
2. Separate males from females and allocate target individuals to experimental containers (refer to **Note** **39**).
3. Store vials at 25 °C, 65 % humidity, 12:12 h light–dark.
4. Transfer to fresh food every 2–3 days. Conduct egg counts as described in Subheading 3.2.
5. At each transfer score deaths and censors until all flies are dead.

#### Protocol Modifications for Measuring Effects of Genetic interventions to Modify Life Span

1. Repeat **steps 1**–**3** from Subheading 3.2 (*see* **Note** **40**).
2. Separate males from females and allocate target individuals to experimental containers (refer to **Note** **41**).
3. Store vials at 25 °C, 65 % humidity, 12:12 h light–dark.
4. Transfer to fresh food every other day. Conduct egg counts as described in Subheading 3.2.
5. For each transfer, score deaths and censors until all flies are dead.

#### Modifications for Measuring the Effects of Pharmacological interventions to Alter Life Span

1. Repeat **steps 1**–**3** from Subheading 3.2
2. Separate males from females and allocate target individuals to experimental containers (refer to **Note** **42**).
3. Store vials at 25 °C, 65 % humidity, 12:12 h light–dark.
4. Transfer to fresh food every other day. Conduct egg counts as described in Subheading 3.2.
5. For each transfer, score deaths and censors until all flies are dead

### Data Handling

1. For each Life Spanecord the date on which the experiment started, the Genotype, and conditions used in the experimental setup as well as any notes about the experimental setup that will modify or help interpretation of the outcomes. For consensus guidelines on what constitutes the minimal information to be recorded for Life span experiments, *see* ref. 17.
2. Throughout the experiment, record deaths and censors for each vial for each day on which they were observed (*see* **Note** **43**).
3. These data can be used to generate life span curves for comparison using Life spantandard life table analyses [18].
4. An important recent advance has been the publication of an openly available Database for storing Life span data, called SurvCurv [17, 19, 20]. Users can upload data for secure storage as well as use an array of statistical tools to analyze the experimental outcomes. Additional tools available on the site allow the Life span to be compared to others in the Database and so can be used to aid further biological discoveries.

## Notes

1. Automatic cookers with built-in stirrers like the Joni Multimix (Joni Foodline) are useful for standardizing large volume cooks.
2. We use red grape juice that is designed for use in home wine production. Many laboratories use apple juice.
3. Our simple recipe of sugar and whole yeast lysate provides nutrition for optimal Development and Life span. Many alternatives exist, but not all are optimal (see supplement to [5]). Most recently, we have described a standardized holidic Diet that contains all necessary nutrients to support long life [21]. It is important to note that our recipes contain the nutritional complement of whole yeast preparations, which cannot simply be replaced by water soluble yeast extract that does not support long life [7].
4. In a relatively cool climate where room temperatures do not exceed 22 °C, this can be overnight. If medium shrinks in vials and pulls away from the edges, this is a sign of over-drying.
5. Housing trays of vials/bottles in pillowslips as they cool is a useful way to protect them from stray flies.
6. Alternatives to cotton wool balls exist: for example polyurethane foam plugs (available from www.drosophilacenter.com) are mite resistant, retain their structure and are reusable after washing.
7. To avoid the need to plug hundreds of vials before storage, it is possible to seal trays with Glad^®^ Press’n Seal. If doing so, it is extremely important to ensure the seal is sound, there are no holes in the plastic film and all vials are covered to avoid both contamination and food from excessive drying when cooled.
8. A useful resource for equipment suitable for use in *Drosophila* research is the supplier: www.flystuff.com (a division of Genesee Scientific).
9. In situations where small numbers of parental flies are used for egg lays, it is more space and resource efficient to use small (~5 cm diameter) petri dishes and cages.
10. We use tips from StarLab (Cat Number: E1011-5100), but it is also possible to cut back a standard pipette tip a few mm to make a wide opening.
11. Backcrossing for six generations is, in almost all cases, sufficient to eliminate the confounding effects of genetic background. It should be noted that this should be performed to each laboratory’s own genetic stocks since even inbred lines with the same name will differ between laboratories [22].
12. If not experienced with fly handling, replacing the egg laying plate may require flies to be lightly anesthetized with CO_2_.
13. It is important not to use too much live yeast for the egg collection plate as it interferes with egg collection. Nor do you want to use too little such that the yeast supply is exhausted. Aiming to have a small amount left at the egg lay is ideal. A cage of ~300 flies will consume ~1 tsp of live yeast overnight.
14. While overnight egg lays produce adequate synchronization for most Life span experiments, this egg collection window can be reduced.
15. To time the emergence of adults so that it falls on a weekday, transfer embryos to fresh food for Development on a Friday. Emerging flies will be available on Monday, 9.5 days later.
16. If egg yield is a problem, the same parents can be used for an additional lay on a fresh plate containing live yeast.
17. A dense mass of eggs yields ~300 adult flies.
18. Before incubating, it is best to remove any leftover yeast paste from the egg laying plate as emerging larvae will burrow into it, making them hard to collect.
19. This is the smallest of three larval stages.
20. Before transferring larvae into fresh media, make a dent in the food to make it easier to wipe off the larvae against a slope of food.
21. For practical reasons, collecting larvae by picking is more manageable using 30 ml vials containing ~7 ml of SY food. Overcrowding can be avoided with 30–50 larvae.
22. At the larval densities recommended in this protocol, larvae will migrate to the cotton wool to pupate. If the container is not tightly plugged, the larvae will escape from the bottle.
23. In order to collect virgin flies, check bottles at 9 days after egg transfer and clear any flies that have emerged. Check the bottle every ~4 h for newly emerged flies—these will all be virgins. Transfer virgin flies to a cold, clean bottle on ice and sort males from females while they remain in a chill coma. CO_2_ should be avoided as the fly’s cuticle is immature, and exposure to the gas can lead to adverse effects on Life span Genders can only be distinguished with the use of a dissecting microscope to examine the genitalia.
24. It is best to transfer newly emerged flies without using CO_2_.
25. In order to anesthetize a whole bottle of flies rapidly, fill a fresh empty (without food), dry bottle with CO_2_ and transfer all flies into it. When sorting anesthetized flies, work on a perforated plate through which a stream of CO_2_ is passing. To avoid desiccating the flies, it is ideal to bubble the CO_2_ through water before it reaches the flies.
26. When allocating experimental flies to treatment, ensure the representation of individuals from each rearing container is balanced between experimental treatments.
27. For standard experiments, 100–150 flies per treatment housed as groups of 10–15 flies per vial are a manageable size. The lifetime outcome for all flies per vial should be recorded. Although the population across all vials for a treatment is treated as one during analysis, this approach allows the performance of individual vials to be revisited if outliers are suspected.
28. Experimental conditions can be blinded to the experimenter at this stage.
29. Fly Life span varies with the size of their housing. Experiments comparing the life spans of flies kept in 25 ml versus 500 ml flasks, but at a standard density per container volume, found that life span was significantly shorter in the larger Life spanume flasks. Shorter life span was associated with higher levels of flying activity [23].
30. There is a range of densities of flies per container that is optimum for life span. In a series of 30 Life span vials [24] found the Life spanoptimum to be for 2–15 flies per container and above this density saw a decrease in life span for each increase in population density.
31. As flies age and become frail, they have an increased risk of falling and becoming stuck in their food. They will also spend more time at the base of the vial. Life spang the vials on their sides during life spans, so the food is a vertical surface at one end of the vial rather than the floor, reduces the risk of these accidental causes of death.
32. Depending on experience with handling *Drosophila*, during the first 2 weeks of a life span experiment the flies may be too fast to transfer between Life spanwithout light anesthesia. Males are more active and move more quickly than females and so are more likely to escape without anesthesia. With practice and good technique, it should be possible to transfer flies without CO_2_.
33. When transferring flies, record deaths and censors (accidental deaths or escapees). Remember to note any dead flies transferred to new vials so that they can be deducted from the number of deaths recorded during the next transfer.
34. Some flies are bang sensitive, and appear to become mores so with age. These can appear dead during the disturbance of transfer. To avoid counting these as dead, first scan vials for deaths, then transfer all vials to new food and after that, check vials for dead flies transferred to fresh media.
35. To reduce labor and use of resources, it is possible to reuse the cotton ball that stoppers a vial by transferring it to the fresh vial to which flies are transferred. However, over time the cotton balls will deteriorate and so it is best to replace them at least once a month.
36. A sample timetable for transferring flies and counting eggs can be: transfer flies to fresh food on Mon, Wed and Fri afternoons, count eggs on Tuesady and Thursday mornings.
37. Record the time the flies go on the food and the time at which eggs in the vial were counted.
38. For young flies, there may be a lot of eggs. If there are too many to count with 10–15 flies in a vial, consider setting up a parallel cohort of flies with fewer females per vial. These flies will not contribute information to the life span experiment (as their density of housing is different from those in the experiment) and they can be discarded when egg counting is complete.
39. To control for rearing conditions in larger experiments, it is good practice to use one rearing bottle per experimental replicate vial. For example: for an experiment with 15 vials of flies per food type, generate 15 rearing bottles; when allocating flies to treatment, anesthetize rearing bottle 1 and allocate 10 flies to experimental vial number 1 for treatment A, then B, C, D and so on until all treatments have one vial populated from the same rearing bottle. Repeat this system with a new rearing bottle for the second replicate vial for each treatment.
40. Some genetically modified lines will have altered (usually longer) development time. In order to synchronize the start of the Life span experiment, initiate the parental crosses for the retarded lines so that egg collection is performed before that of the non-delayed lines. To buffer against slight variations in the delay, it is best to rear multiple batches of the experimental generation, derived from consecutive days of egg laying. This way it will be possible to collect flies from all lines that have emerged within 24 h of each other.
41. It may not be possible to control for rearing conditions when using different genotypes in the same way as for single Genotypes between multiple experimental foods. However, if the genetic scheme allows, it may be possible to use sibling flies as controls for experimental flies. Alternatively, it may be possible to rear multiple genotypes in a single rearing container. However, it is important to determine first that these larval conditions to not interact with Life span outcomes.
42. To control for rearing conditions, use the protocol employed for testing the effects of multiple food types on one Genotype (**Note** 40).
43. Each laboratory has its own Method of recording and plotting these data. An Excel sheet used in our laboratories can be found at: http://piperlab.org/resources/. More sophisticated and automated packages can be found through the Pletcher laboratory (*see* ref. 25 and associated URLs).

## References

[1] Kenyon CJ (2010). The genetics of ageing. Nature.

[2] Partridge, L., Alic, N., Bjedov, I., and Piper, M.D. (2011). Ageing in Drosophila: the role of the insulin/Igf and TOR signalling network. Exp. Gerontol. 46, 376–381. 10.1016/j.exger.2010.09.003 PMC3087113 20849947

[3] Niccoli T, Partridge L (2012). Ageing as a risk factor for disease. Curr Biol.

[4] Loeb J, Northrop JH (1916). Is there a temperature coefficient for the duration of
life?. Proc Natl Acad Sci U S A.

[5] Piper MD, Partridge L (2007). Dietary restriction in Drosophila: delayed aging or
experimental artefact?. PLoS Genet.

[6] Partridge L, Gems D (2007). Benchmarks for ageing studies. Nature.

[7] Bass TM, Grandison RS, Wong R, Martinez P, Partridge L, Piper MD (2007). Optimization of dietary restriction protocols in
Drosophila. J Gerontol A Biol Sci Med Sci.

[8] Zwaan BJ, Bijlsma R, Hoekstra RF (1991). On the developmental theory of ageing. I. Starvation
resistance and longevity in Drosophila melanogaster in relation to pre-adult
breeding conditions. Heredity.

[9] Pearl R (1921). The biology of death--VI. Experimental studies on the
duration of life. Sci Mon.

[10] Partridge L, Andrews R (1985). The effect of reproductive activity on the longevity of
male Drosophila melanogaster is not caused by an acceleration of
ageing. J Insect Physiol.

[11] Partridge L, Farquhar M (1981). Sexual activity reduces lifespan of male
fruitflies. Nature.

[12] Partridge L, Fowler K, Trevitt S, Sharp W (1986). An examination of the effects of males on the survival
and egg-production rates of female Drosophila melanogaster. J Insect Physiol.

[13] Partridge L, Green A, Fowler K (1987) Effects of egg - production and of exposure to males on female survival in Drosophila melanogaster. J Insect Physiol 33:745–749

[14] Sgro CM, Partridge L (1999). A delayed wave of death from reproduction in
Drosophila. Science.

[15] Chapman T, Partridge L (1996). Female fitness in Drosophila melanogaster: an
interaction between the effect of nutrition and of encounter rate with
males. Proc Biol Sci.

[16] Wigby, S., Slack, C., Grönke, S., Martinez, P., Calboli, F., Chapman, T., and Partridge, L. (2011). Insulin signalling regulates remating in female Drosophila. Proc Roy Soc B 278, 424–431. 10.1098/rspb.2010.1390 PMC3013413 20739318

[17] Ziehm, M., Ivanov, D.K., Bhat, A., Partridge, L., and Thornton, J.M. (2015) SurvCurv database and online survival analysis platform update. Bioinformatics 31(23):3878–80 10.1093/bioinformatics/btv463 PMC4653391 26249811

[18] Lee E, Wang J (2003). Statistical methods for survival data analysis.

[19] Ziehm M, Thornton JM (2013). Unlocking the potential of survival data for model
organisms through a new database and online analysis platform:
SurvCurv. Aging Cell.

[20] Ziehm M, Piper MD, Thornton JM (2013). Analysing variation in Drosophila aging across
independent experimental studies: a meta-analysis of survival data. Aging Cell.

[21] Piper MDW, Blanc E, Leitão-Gonçalves R, Yang M, He X, Linford NJ, Hoddinott MP, Hopfen C, Soultoukis GA, Niemeyer C, Kerr F, Pletcher SD, Ribeiro C, Partridge L (2014) A holidic medium for Drosophila melanogaster. Nat Methods 11(1):100–5 10.1038/nmeth.2731 PMC3877687 24240321

[22] Berger J, Suzuki T, Senti K-A, Stubbs J, Schaffner G, Dickson BJ (2001) Genetic mapping with SNP markers in Drosophila. Nat Genet 29(4):475–81 10.1038/ng773 11726933

[23] 70Magwere, T., Pamplona, R., Miwa, S., Martinez-Diaz, P., Portero-Otin, M., Brand, M., and Partridge, L. (2006). Flight activity, mortality rates, and lipoxidative damage in Drosophila. Journals Gerontology 61(2): 136–145 10.1093/gerona/61.2.136 16510857

[24] Pearl, R (1928). Experiments on longevity. The Quarterly Review of Biology 3(3):391–407

[25] Linford, N., Bilgir, C., Ro, J., and Pletcher, S. (2013). Measurement of lifespan in Drosophila melanogaster. J Vis Exp (71), e50068, doi:10.3791/50068 10.3791/50068 PMC3582515 23328955

